# Emergence of the invasive Asian bush mosquito *Aedes* (*Hulecoeteomyia*) *japonicus* (Theobald, 1901) in the Czech Republic

**DOI:** 10.1186/s13071-022-05332-5

**Published:** 2022-07-11

**Authors:** Jakub Vojtíšek, Nele Janssen, Silvie Šikutová, Oldřich Šebesta, Helge Kampen, Ivo Rudolf

**Affiliations:** 1 Institute of Vertebrate Biology, The Czech Academy of Sciences, Brno, Czech Republic; 2 Department of Experimental Biology, Masaryk University, Brno, Czech Republic; 3 Friedrich-Loeffler-Institut, Federal Research Institute for Animal Health, Insel Riems, Greifswald, Germany

**Keywords:** *Aedes japonicus*, Central Europe, Introduction, Invasive species, Surveillance, Vector

## Abstract

**Background:**

*Aedes japonicus* is a mosquito species native to North-East Asia that was first found established outside its original geographic distribution range in 1998 and has since spread massively through North America and Europe. In the Czech Republic, the species was not reported before 2021.

**Methods:**

*Aedes* invasive mosquitoes (AIM) are routinely surveyed in the Czech Republic by ovitrapping at potential entry ports. This surveillance is supported by appeals to the population to report uncommon mosquitoes. The submission of an *Ae. japonicus* specimen by a citizen in 2021 was followed by local search for aquatic mosquito stages in the submitter’s garden and short-term adult monitoring with encephalitis virus surveillance (EVS) traps in its surroundings. Collected *Ae. japonicus* specimens were subjected to *nad*4 haplotype and microsatellite analyses.

**Results:**

*Aedes japonicus* was detected for the first time in the Czech Republic in 2021. Aquatic stages and adults were collected in Prachatice, close to the Czech-German border, and eggs in Mikulov, on the Czech-Austrian border. Morphological identification was confirmed by molecular taxonomy. Genetic analysis of specimens and comparison of genetic data with those of other European populations, particularly from Germany, showed the Prachatice specimens to be most closely related to a German population. The Mikulov specimens were more distantly related to those, with no close relatives identifiable.

**Conclusions:**

*Aedes japonicus* is already widely distributed in Germany and Austria, two countries neighbouring the Czech Republic, and continues to spread rapidly in Central Europe. It must therefore be assumed that the species is already present at more than the two described localities in the Czech Republic and will further spread in this country. These findings highlight the need for more comprehensive AIM surveillance in the Czech Republic.

**Graphical Abstract:**

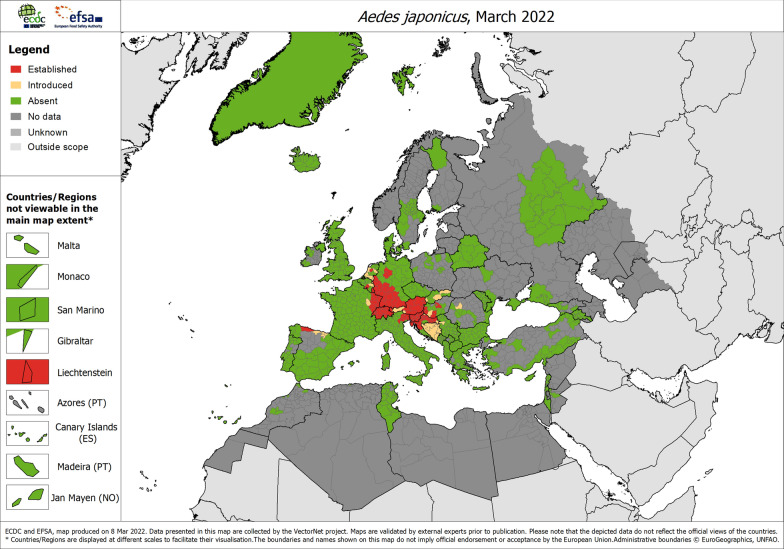

**Supplementary Information:**

The online version contains supplementary material available at 10.1186/s13071-022-05332-5.

## Background

The presence of four *Aedes* invasive mosquito (AIM) species in Europe is consistently noted: *Aedes aegypti*, *Aedes albopictus*, *Aedes japonicus* and *Aedes koreicus* have been introduced into many European countries and have succeeded in establishing in several of them. Rather than being a nuisance, these mosquito species may pose a serious threat to public health by their capability of transmitting arboviruses such as dengue, chikungunya, yellow fever and Zika viruses. The intercontinental spread of these AIMs is facilitated by global trade [1] and is attributable to their drought-resistant eggs [2]. Establishment and further dispersal in the new geographical regions are possible when climatic and environmental conditions are adequate. In Central Europe, *Ae. japonicus* and *Ae. koreicus* find similar (moderate) climatic conditions as in their original Asian distribution range, while the thermophilic species *Ae. albopictus* increasingly adapts to moderate temperatures and takes advantage of climate warming.

*Aedes* (*Hulecoeteomyia*) *japonicus* (Theobald 1901), also known as the Japanese bush mosquito or Asian rock pool mosquito, is native to North-East Asia [3]. Its first finding in Europe was reported in 2000 from north-western France, where two larvae were collected in car tyres [4]. Subsequently, the species was found in Belgium in 2002 as larvae and in 2004 as adults [5]. Later reports describe occurrences in Switzerland and Germany in 2008, Austria and Slovenia in 2011, and the Netherlands and Hungary in 2012 [6–8]. In 2013, the establishment of *Ae. japonicus* was confirmed in France [9], and in the same year, the emergence of this mosquito was reported from Croatia [10]. Its spread continued in 2015 to Liechtenstein and Italy, in 2017 to Bosnia and Herzegovina, and in 2018 to Serbia, Spain and Luxembourg [11–14]. The most recent report of *Ae. japonicus* introduction was added from Romania for 2020 [15].

Unlike *Ae. albopictus*, which is exclusively spread by human mediation, *Ae. japonicus* spreads to new areas by active dispersal, too [16, 17], with spreading corridors mainly being the borders between rural and urban habitats, such as suburban gardens, vineyards, small patches of forest adjacent to fields or forest edges with streams [12, 18, 19]. Adults usually live in forests but migrate to gardens or cemeteries to lay eggs, where they find more potential breeding sites, i.e. artificial containers with stagnant water [20]. In the United States, larvae of *Ae. japonicus* are commonly found in rock pools, similar to their native range, but in Europe they have also been collected from tree holes and a variety of man-made containers such as barrels, tyres, bathtubs or flower vases [12, 13, 21]. *Aedes japonicus* typically seeks larger containers than other container-breeding AIMs [19, 21]. In Switzerland and Germany, the species *Aedes geniculatus* and *Anopheles plumbeus* use similar breeding sites as *Ae. japonicus* [18, 20].

Moreover, *Ae. japonicus* is well adapted to cold winters and snow. Its larvae have been found in water as cold as 4 °C [16]. Its occurrence above 700 m above sea level (a.s.l.) in south-eastern Europe [12] and at 1200 m a.s.l. in the German Black Forest [22] suggests that crossing mountains should not be very problematic for this species. In the southern Appalachians in the United States, *Ae. japonicus* was the only container-breeding mosquito species found above 1400 m a.s.l. [21].

*Aedes japonicus* feeds on a variety of hosts, but appears to be less anthropophilic than other AIMs [23].

There is no evidence of pathogen transmission by *Ae. japonicus* under natural conditions. Therefore, this species is considered less important as a public health vector than *Ae. aegypti* or *Ae. albopictus*. However, genetic material of West Nile virus (WNV) has been demonstrated several times in *Ae. japonicus* collected in the field in the USA [24, 25]. Furthermore, in laboratory studies, this mosquito shows vector competence for eastern equine encephalitis, La Crosse, St. Louis encephalitis, Rift Valley fever, Usutu, chikungunya, dengue and Zika viruses [26–31] in addition to WNV [25], as well as for the nematodes *Dirofilaria repens* and *Dirofilaria immitis* [32].

We here describe the first encounters with *Ae. japonicus* mosquitoes in the Czech Republic, which took place in 2021, independently in two areas more than 190 km apart. One finding locality was localised close to the border with Germany and the second on the border with Austria.

## Methods

### Study sites and mosquito collection

In the evening hours of 15 June 2021, a citizen of the town of Prachatice in South Bohemia caught a strangely coloured adult female mosquito in his house. He sent five pictures of this mosquito to our laboratory for preliminary identification. Since it was not possible on these to distinguish between *Ae. japonicus* and *Ae. koreicus*, the pictures were followed by the physical specimen for accurate identification. On 9 July, two additional adult mosquitoes of the same appearance (one male, one female) were captured by the citizen at the same location.

From 11 to 13 August, the garden of the reporting resident and its surroundings were checked for AIMs by dipping water containers for aquatic mosquito stages and trapping adult mosquitoes by encephalitis virus surveillance (EVS) traps equipped with dry ice as an attractant. Sampling of aquatic mosquito stages was done repeatedly on 11 and 12 August from three dark green plastic rainwater collection barrels, that stood immediately against the house wall, each holding approximately 300–400 L of water and having a water surface area of about 0.8 m^2^. From 11 to 12 August, eight EVS traps were operated within 100 m and another seven traps within 1.5 km from the submitter’s house. Two EVS traps were placed even further away (about 3.5 km). The traps were located at shady places near the gardens of family houses, in an allotment area and on the edge of a forest. Three of the traps in the 100 m diameter were placed about 20 m from the rainwater barrels, another two about 50 m and three about 80 m. One of the three traps within 20 m distance was equipped with sweaty socks as an additional attractant. All traps were run overnight. On 12 August, one trap was kept working all day next to the rainwater barrels. From the evening of 12 August to the morning of 13 August, a total of 14 EVS traps were operated in close vicinity (100 m) of the rainwater barrels in the submitter’s garden.

**Fig. 1:**
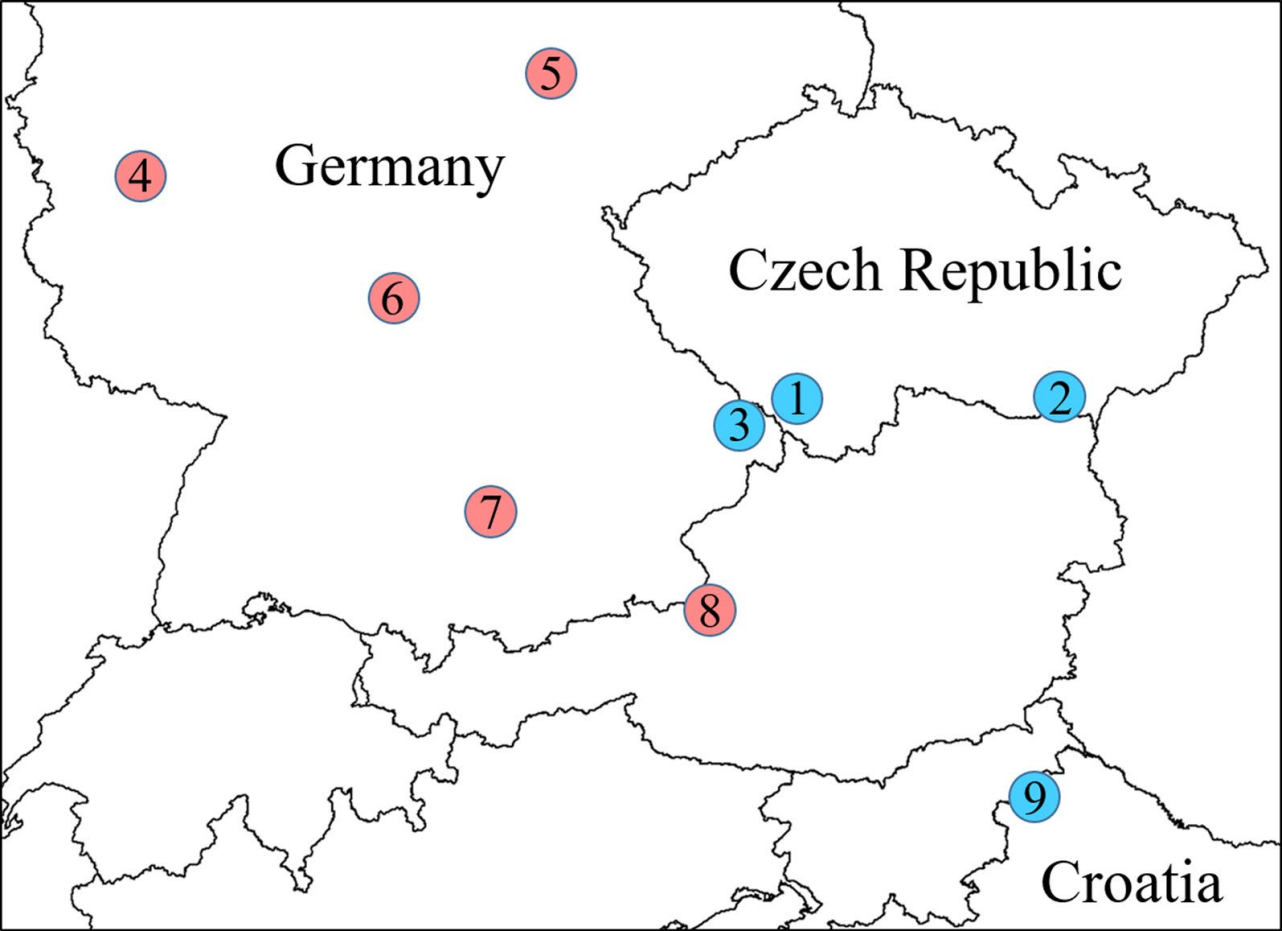
Location of *Ae. japonicus* collection sites in the Czech Republic (nos. 1, 2) and of *Ae. japonicus* populations genetically compared with them (nos. 3–9). Names of locations are provided in Table 1; colours of dots refer to the two genetic clusters as shown in Fig. 3

The city of Prachatice (Fig. 1) is situated in the foothills of the Bohemian Forest and borders its protected landscape area, which includes the Bohemian Forest National Park. The town is located in a valley basin with a flowing stream at an altitude of 561 m a.s.l. In the surrounding area, there are many suburbs with gardens and gardening colonies. The landscape is hilly and consists of forest and meadows rather than agricultural land. About 25 km to the south-east runs the German-Czech border, with *Ae. japonicus* occurrence reported not far from the border on the German side (Kampen & Werner, unpublished data).

Ovitrapping was done in the framework of a long-term surveillance programme focusing on the occurrence of *Ae. albopictus* at the border between the Czech Republic and Austria [33, 34]. The site ‘Mikulov 2’ (Fig. 1) is one of three parking lots in the outskirts of the town of Mikulov included in the monitoring programme. This place is located between the motorway A5 on the Austrian side and the motorway 52 to Brno on the Czech side and is used by lorry drivers as a stopover. For a detailed description of the surveillance methodology by ovitrapping, see Rudolf et al. [34].

### Rearing and identification of mosquitoes

Oviposition supports with eggs from ovitraps were brought to the laboratory where the eggs were flooded in jars with stale tap water for the larvae to hatch. Hatched larvae were fed ground fish food (Tetra GmbH, Melle, Germany). Larvae from the rainwater barrels were transferred to jars together with original barrel water, the organic compounds of which served as larval food during further development. Jars were covered with nets until emergence of adults, which were collected with a battery-powered aspirator (Hausherr’s Machine Works, Toms River, NJ, USA) and killed by freezing at −20 °C for at least 24 h.

Adults were morphologically identified using the determination key provided in Becker et al. [35]. Morphological determination was confirmed genetically on one specimen from Prachatice and two specimens from Mikulov (adults). For this purpose, one leg per mosquito specimen was individually homogenised in 150 μl sterile demineralised water with sterile 5 mm steel beads at 30 Hz for 60 s in a TissueLyser II (Qiagen, Hilden, Germany). Genomic DNA was extracted from 100 μl of the homogenates using the QIAamp DNA Mini Kit (Qiagen) according to the manufacturer’s protocol. The DNA was eluted in 80 µl elution buffer and stored at −20 °C until further processing.

Partial segments of the mitochondrial cytochrome c oxidase subunit 1 (*cox*1) [36, 37] and of the NADH dehydrogenase subunit 4 (*nad*4) genes were amplified for mosquito identification [38] using Combi PPP Master Mix (Top-Bio, Vestec, Czech Republic) following published protocols [39]. Polymerase chain reaction (PCR) products were sequenced bidirectionally according to Janssen et al. [40] and the sequences obtained blasted to the GenBank sequence library (https://blast.ncbi.nlm.nih.gov).

### Population genetic analysis

To obtain clues on the origin of the Czech *Ae. japonicus* samples and their relationships to conspecific populations in Europe, population genetic analyses were performed on 20 individuals from Prachatice, 14 individuals from Mikulov and 20 individuals from Grafenau, the closest German place to the Czech border and Prachatice known to be colonised by *Ae. japonicus* (beeline distance between Grafenau and Prachatice ca. 50 km). The data obtained from these three locations were compared with data from previously investigated *Ae. japonicus* populations from other areas in Germany and one location in Croatia (Table 1). Analysis was performed on *nad*4 haplotypes and microsatellites. For the latter, seven polymorphic loci (OJ5, OJ10, OJ70, OJ85, OJ100, OJ187, OJ338) were genotyped as described by Janssen et al. (2019). The results of the microsatellite analysis were interpreted with Geneious Prime version 2021.0.1 (Geneious Biomatters, Auckland, New Zealand), subjected to a Bayesian cluster analysis using STRUCTURE [41] and evaluated with STRUCTURE HARVESTER [42]. Furthermore, based on Nei’s genetic distance and pairwise *F*_*ST*_ values, a principal coordinate analysis (PCoA) was performed.

**Table 1:** Origin and number of processed specimens from current and previously investigated populations of *Ae. japonicus* in Europe

Population	Country	Collection year	Number of specimens processed	Reference
(1) Prachatice	Czech Republic	2021	20	This study
(2) Mikulov	Czech Republic	2021	14	This study
(3) Grafenau	Germany	2019	20	This study
(4) Linz	Germany	2017	24	[40]
(5) Burgscheidungen	Germany	2017	20	[40]
(6) Wurzburg	Germany	2017	30	[40]
(7) Augsburg	Germany	2017	19	[40]
(8) Berchtesgaden	Germany	2017	13	[40]
(9) Macelj	Croatia	2017	15	[40]

## Results

### Mosquito collection and identification

**Fig. 2:**
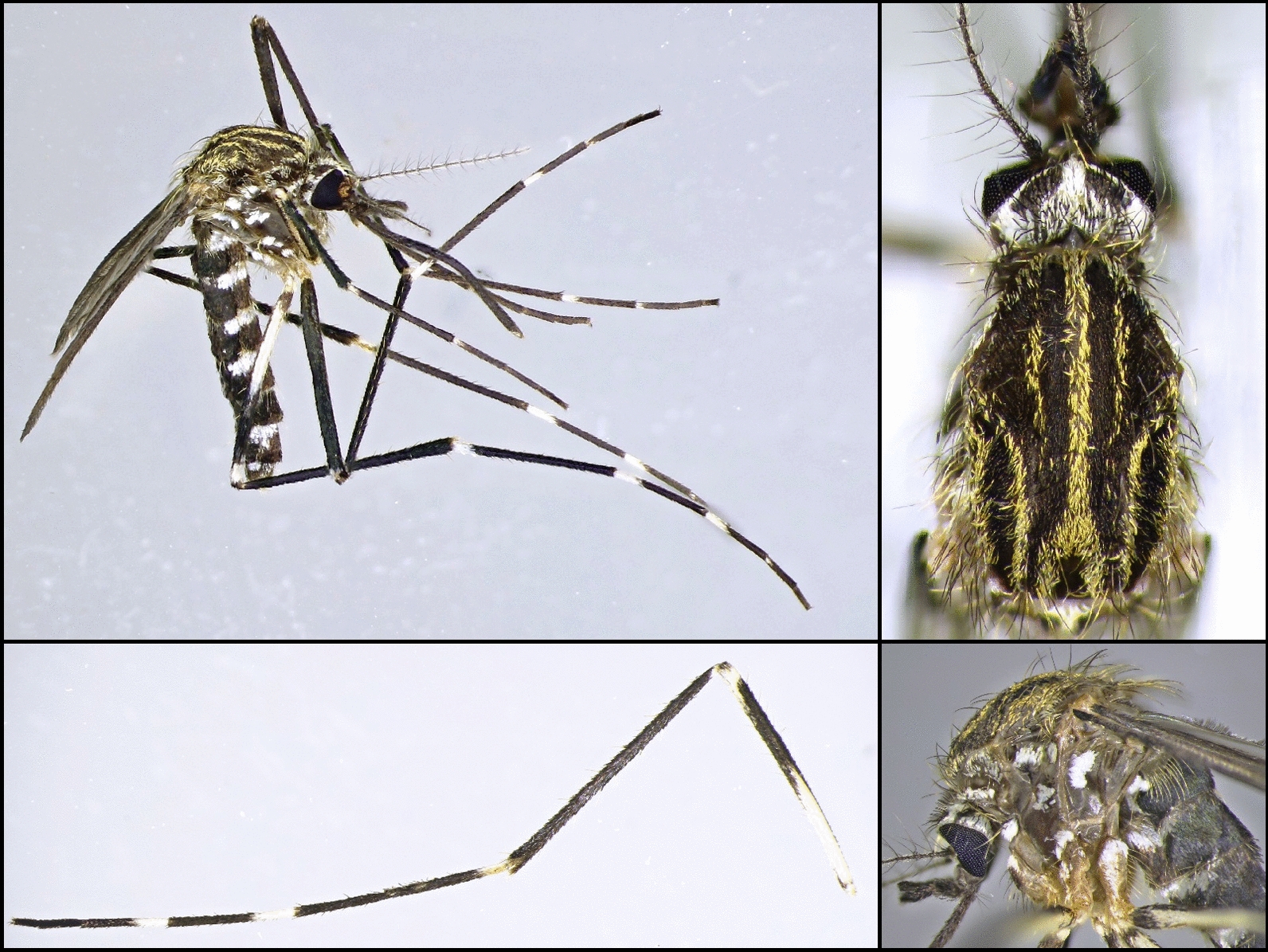
The very first *Ae. japonicus* mosquito detected in the Czech Republic, submitted from Prachatice. **a** Overall view of the specimen. **b** Five stripes of golden scales on the scutum. **c** Hind leg with no white rings on tarsal segments IV and V (as opposed to *Ae. koreicus*). **d** Lateral view of thorax

The first adult female mosquito from the Prachatice locality was identified as *Ae. japonicus* on the basis of characteristic morphological features—scutal stripes, scales on the sides of the thorax, tergite scale pattern and hind leg colouration (Fig. 2). Molecular analysis of that specimen revealed 99.86% similarity of the *cox*1 partial sequence (GenBank accession no. OM307664) to *Ae. japonicus* GenBank accession no. KF211505 from Germany and 99.35% similarity of the obtained *nad*4 partial sequence (GenBank accession no. OM307666) to *Ae. japonicus* GenBank entry AF305879 from Germany.

Adults developed from several dozen larvae and pupae collected from the three rainwater barrels (out of hundreds present) were also morphologically identified as *Ae. japonicus*. Other mosquito species detected in the barrels belonged to the *Culex pipiens* and *Anopheles maculipennis* complexes.

No *Ae. japonicus* was collected in the EVS traps during the first night of trapping in Prachatice, but one *Ae. japonicus* female was captured during the second night with the EVS trap equipped with sweaty socks. All other EVS traps remained negative for *Ae. japonicus*. However, *Cx. pipiens* complex females and some *Aedes vexans* specimens were collected by the EVS traps within the 100 m radius, and several individuals of *Ae. geniculatus* and *An. plumbeus* were collected from EVS traps placed on the forest edge. One EVS trap placed in a garden colony contained an *An. maculipennis* complex female.

Thirty-six morphologically identified adult *Ae. japonicus* (25 females, 11 males) emerged between 28 and 30 August 2021 from ovitrap egg collections carried out from 10 to 18 August 2021 at ‘Mikulov 2’. The sequences of the two tested *Ae. japonicus* individuals were identical (GenBank accession nos. OM307665 for *cox*1 and OM307667 for *nad*4) and shared 99.58% nucleotide homology with a *cox*1 sequence from an isolate from Germany (GenBank accession number KF211505) and 99.14% nucleotide homology with a *nad*4 sequence from an isolate from the USA (GenBank accession number AF305879).

### Population genetic analysis

*Nad*4 haplotype analysis was performed on 54 specimens from Prachatice, Mikulov and Grafenau (south-eastern Germany). The alignment indicated 21 variable nucleotide positions, leading to four different *nad*4 haplotypes: H1, H3, H9 and H21 (Table 2). Six individuals were characterised by heteroplasmy, the presence of different mitochondrial DNA (mtDNA) variants in one organism, as indicated by peaks for two different nucleotides at the same position in the sequencing electropherogram.

**Table 2:** *Nad*4 haplotypes detected in *Ae. japonicus*

Population	Number of individuals examined	No. of heteroplasmic individuals	*nad*4 haplotypes			
			H1	H3	H9	H21
Prachatice	20	5	5	2	3	5
Mikulov	14	0	0	0	13	1
Grafenau	20	1	14	0	5	0

**Fig. 3:**
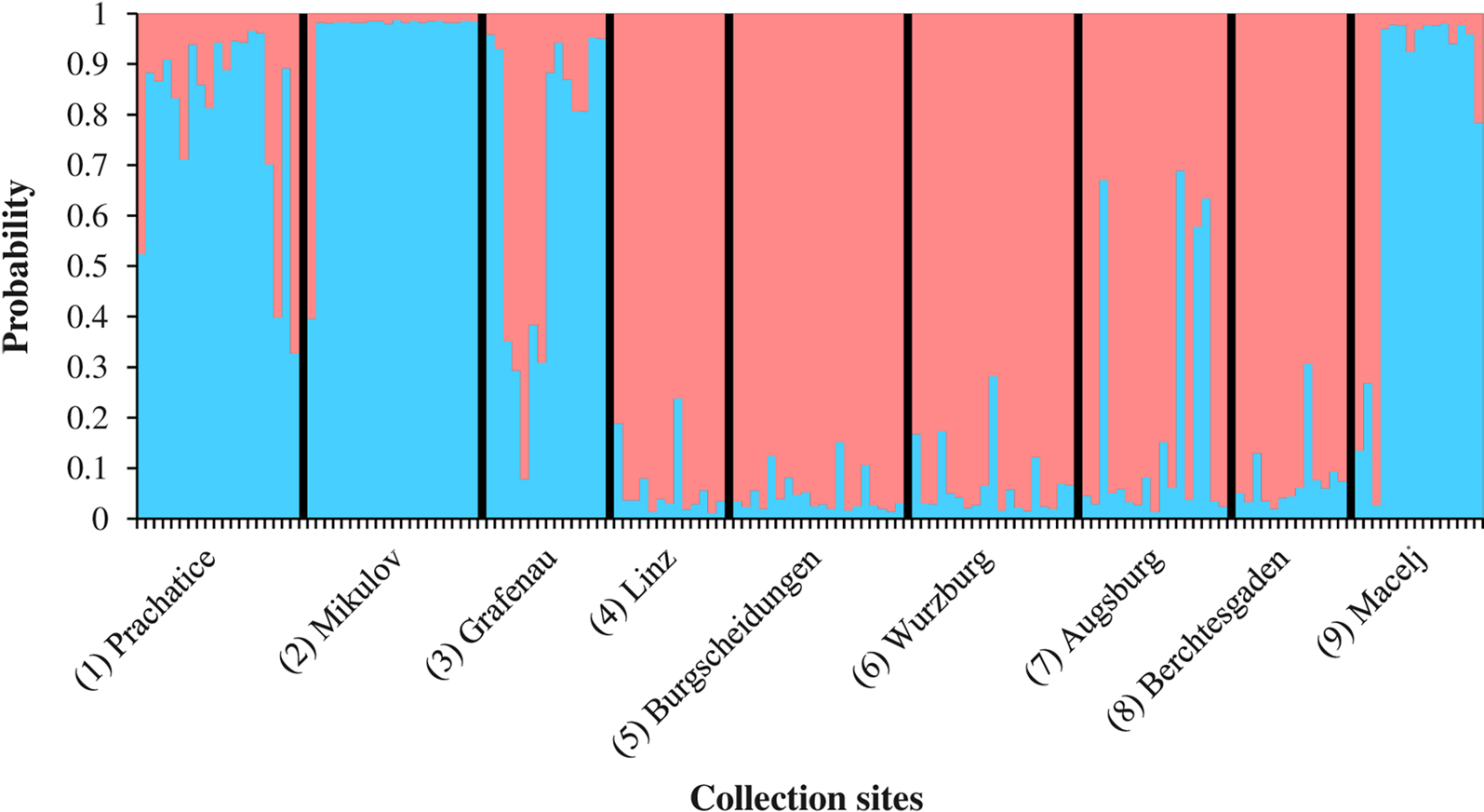
Results of microsatellite multilocus genotyping for *k* = 2 (∆ = 160.08), with each bar representing a single individual and the different colours representing the probability of that individual belonging to the corresponding genetic cluster

Analysable microsatellite data were obtained for 53 samples from the two Czech populations and the German population from Grafenau. For comparison, data available from previous studies on German and Croatian *Ae. japonicus* populations were included in the analysis (Additional file 1: Table S1). The Bayesian cluster analysis showed the highest probability for the existence of two genetic clusters among the tested populations (*k* = 2; ∆ = 160.08). According to this analysis, the populations from Prachatice, Mikulov, Grafenau and Macelj (north-western Croatia) have a high probability of belonging to the same microsatellite genetic cluster 1 (blue colour), whereas the previously investigated populations from Germany rather appear to belong to genetic cluster 2 (red colour) (Fig. 3). Despite the same principal genetic cluster 1, the Mikulov population seems to be somewhat different from the Prachatice, Grafenau and Macelj populations.

**Fig. 4:**
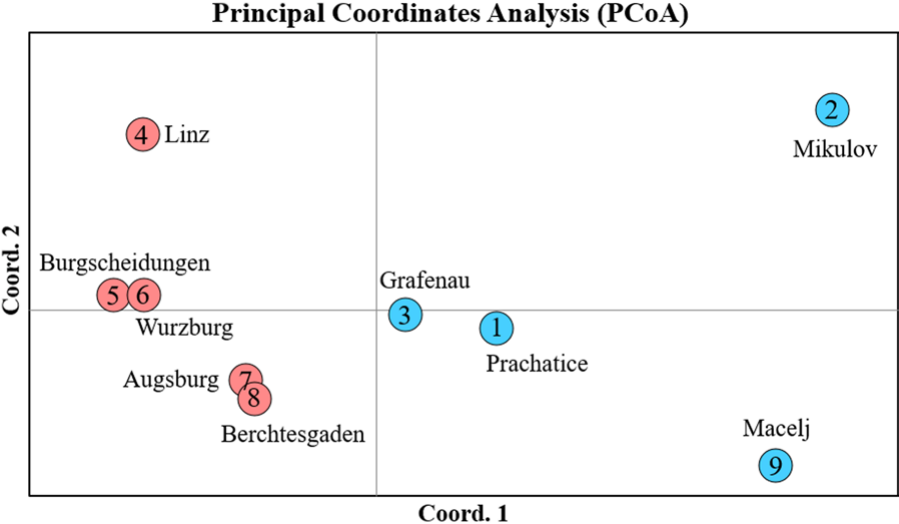
Principal coordinates analysis (PCoA) plot of pairwise population F_ST_ values for the investigated *Ae. japonicus* populations from the Czech Republic, Germany and Croatia. The colours of the dots (blue and red) represent the principal microsatellite genotype according to Fig. 1

The results of the PCoA, based on F_ST_ values and Nei’s genetic distance of the microsatellite locus data, show a close genetic relatedness of the Prachatice and Grafenau populations. The other included populations are much more distantly related (Fig. 4).

## Discussion

### Emergence of *Ae. japonicus* in the Czech Republic

The Bohemian Forest region in which the town of Prachatice is embedded provides appropriate landscape structures and paths for the spread of *Ae. japonicus* (e.g. more rural than urban area, presence of many deciduous forest patches and occurrence of transition zones between forest and local settlements) [18, 20]. Based on the demonstrated presence in close-by Germany and the genetic data, an origin of the *Ae. japonicus* specimens from Prachatice and a (possibly active) spread of *Ae. japonicus* from Germany can be assumed. This, however, suggests that *Ae. japonicus* is likely to have already been established between Prachatice and the Czech-German border as well and would explain the high abundance of larvae (hundreds) found in the rainwater barrels in the submitter’s garden, which were hardly produced by a single female. Amazingly, no adult specimens were trapped in Prachatice but this could be due to poor trappability of this species by EVS traps [43]. Larval sampling in Prachatice and the area west of it is planned for 2022 to check for further *Ae. japonicus* occurrence.

Locality ‘Mikulov 2’ is a parking lot with the first gas station on the Czech side of the Czech-Austrian border. A few family houses are about 250 m away. The surrounding landscape on both sides of the border consists mainly of open fields with a few small forest patches and vineyards. Although shady transects are missing, it is not clear whether the emergence of *Ae. japonicus* eggs in ‘Mikulov 2’ is attributable to passive transport and introduction via long-distance traffic of a single gravid female along the highway from the south or to active dispersal. The latter is assumed to have taken place with specimens of *Ae. japonicus* found in the framework of the Austrian mosquito monitoring programme in the Lower Austrian district of Gmünd [44], about 10 km from the Czech border and far from an international traffic route.

### Population genetic analysis

The most frequent *nad*4 haplotype found in the *Ae. japonicus* populations from Prachatice, Mikulov and Grafenau was H9. This *nad*4 haplotype is known from several populations in Europe: Belgium, Austria, The Netherlands, Slovenia and Croatia [12, 39, 40, 45–47]. *Nad*4 haplotype H3 was exclusively detected in Prachatice. This haplotype has been found in populations in South Germany, The Netherlands and Bosnia and Herzegovina [12, 45]. A carry-over by mosquito displacement from those populations to the Czech Republic is possible. The same is true for *nad*4 haplotype H21, as found in both Czech populations in this study, which had previously been detected in South Germany and Bosnia and Herzegovina [12, 45]. In summary, since Prachatice is represented by all four haplotypes found in this study and both Mikulov and Grafenau by two each, but different ones (Table 2), the haplotype analysis is not informative enough to deduce relatedness of the tested populations and displacement/migration routes.

With regard to the results of the microsatellite analysis and the PCoA (Figs. 3, 4), the spatially close collection sites Prachatice and Grafenau show high genetic relatedness, with the second next closest relationship to the populations from Macelj (microsatellite multilocus genotype) or Augsburg and Berchtesgaden (PCoA). A common origin of these populations could be in Austria or Slovenia, where *Ae. japonicus* is widely distributed [48]. The Mikulov population seems to be genetically more different from the other populations tested. A new introduction from a European population not included in the analysis or even from overseas could be the reason.

### Conclusions

Invasive *Ae. japonicus* mosquitoes were reported for the first time in the Czech Republic. Due to the low diversity of *nad*4 haplotypes, the two populations from the Czech Republic as well as that from Grafenau, south-eastern Germany, can be assumed to be relatively young. However, owing to the already wide distribution of *Ae. japonicus* in Europe with ongoing mixture of populations, haplotypes cannot be assigned to certain populations anymore as was the case during the first years of invasion of the western world (e.g. [38, 49, 50]). According to microsatellite and PCoA analyses, the Prachatice population was most likely introduced to the Czech Republic from Germany, while the Mikulov population is at least admixed with genetic material from one (or several) other population(s) of unknown origin.

## Supplementary Information

**Additional file 1: Table S1.** Microsatellite fragment lengths of *Ae. japonicus* populations studied (0: not analysable).

## Data Availability

Data supporting the conclusions of this article are included within the article. Representative DNA sequences have been deposited in the GenBank database under the accession numbers OM307664–OM307667.
